# Facultative mycorrhization in a fern (*Struthiopteris spicant* L. Weiss) is bound to light intensity

**DOI:** 10.1186/s12870-024-04782-6

**Published:** 2024-02-09

**Authors:** Thais Guillen-Otero, Soon-Jae Lee, Dietrich Hertel, Michael Kessler

**Affiliations:** 1 Department of Systematic and Evolutionary Botany, University of Zurich, Zurich, Switzerland; 2 Department of Ecology and Evolution, University of Lausanne, Lausanne, Switzerland; 3 Albrecht von Haller Institute for Plant Sciences, University of Goettingen, Goettingen, Germany

**Keywords:** *Struthiopteris spicant*, Facultative mycorrhizal plant, mycorrhizal status, fern, arbuscular mycorrhizal fungi, ITS, Greenhouse experiment, Light, Nutrients

## Abstract

**Background:**

The establishment of mycorrhizal relationships between a fungus and a plant typically enhances nutrient and water uptake for the latter while securing a carbon source for the fungus. However, under a particular set of environmental conditions, such as low availability of light and abundant nutrients in the soil, the resources invested in the maintenance of the fungi surpass the benefits obtained by the host. In those cases, facultative mycorrhizal plants are capable of surviving without symbiosis. Facultative mycorrhization in ferns has been overlooked until now. The present study measured the response of *Struthiopteris spicant* L. Weiss, and its root-associated fungi to different levels of light and nutrient availability in terms of growth, mycorrhizal presence, and leaf nutrient content. This fern species exhibits a great tolerance to variable light, nutrient, and pH conditions, and it has been found with and without mycorrhizae. We conducted a greenhouse experiment with 80 specimens of *S. spicant* and three factors (Light, Phosphorus, and Nitrogen) resulting in eight treatments.

**Results:**

We found a significant influence of the factor light on fungal community composition, plant biomass, and nutrient accumulation. Departing from a lack of colonization at the initial stage, plants showed a remarkable increment of more than 80% in the arbuscular mycorrhizal fungi (AMF) richness and abundance in their roots when grown under high light conditions, compared with the ones in low light. We also observed an upward trend of C:P and C:N ratios and the above- and belowground biomass production when AMF abundance increased. Furthermore, the compositional analysis of the whole fungal communities associated with *S. spicant* roots revealed clear differences among low-light and high-light treatments.

**Conclusions:**

This study is the first to investigate the importance of light and nutrient availability in determining fern-AMF relationships. We confirmed that *Struthiopteris spicant* is a facultative mycorrhizal plant. The composition and diversity of AMF found in the roots of this fern are strongly influenced by light and less by nutrient conditions. Our study shows that ferns respond very sensitively to changes in environmental factors, leading to shifts in the associated mycorrhizal communities.

**Supplementary Information:**

The online version contains supplementary material available at 10.1186/s12870-024-04782-6.

## Background

Mycorrhizal associations are a widespread ecological strategy for plants to survive under stressful environmental conditions such as droughts, low nutrient availability, and toxic substrates [3, 7]. One of the most ubiquitous and ancient types of mycorrhiza is formed by arbuscular mycorrhizal fungi (AMF) from the phylum Glomeromycota [8, 9, 61]. They are characterized by the development of arbuscules and linear or coiling hyphae within the plant root cortex cells [6, 9, 54]. Plant-AMF interactions have a long evolutionary history demonstrated by genetic and paleontological evidence [55], and they appear to have been a crucial strategy during the early colonization of the land by plants [18, 50]. While a vast number of studies have been conducted on the role of mycorrhizal fungi in seed plants, not much is known about their interactions with early diverging lineages such as lycophytes and ferns [19, 27, 48, 49, 55].

The AMF-plant symbiosis provides advantages for both partners [6, 23]. Fungal colonization usually increases water uptake, productivity, resistance to pathogens, and nutrient availability [7, 19, 52]. The latter is one of the most important functions carried out by mycorrhizal fungi. While phosphorus (P) and nitrogen (N) limit plant growth at a global level [6, 28, 43], fungi hyphae can extend the root surface area, improving the absorption of both elements under restrictive conditions [8, 39].

Plants also experience a cost when hosting AMF, since in exchange for the benefits given, fungi receive 4–20% of the plant’s fixed carbon in the form of carbohydrates and lipids [2, 6, 25]. In theory, plants should favor AMF colonization when their growth is nutrient-limited, and restrict the symbiosis when growth is carbon-limited [21, 58]. However, the existence of multiple approaches to define the cost-benefit analysis highlights the complexity of plant-fungal relationships [58]. The positive response of plants to mycorrhizal colonization depends on light limitation [30, 46], nutrient supply [5, 39], and the host and fungi genotypes [45].

In effect, the nature of the relationship of plants with mycorrhizal fungi is highly variable and can be conditioned by abiotic and biotic factors such as host species and soil characteristics [8, 60]. Three main categories, also known as mycorrhizal status, have been used to describe their capacity to establish mycorrhizal symbioses [7, 9, 54]: obligate mycorrhizal (OM), non-mycorrhizal (NM), and facultative mycorrhizal (FM). OM plants need appropriate fungal partners to survive in natural environments, whereas NM species do not form a symbiosis with fungi throughout their life cycle. On the contrary, FM plants may establish mycorrhizae or not depending on their requirements [9, 54].

Facultative mycorrhization might be seen as an adaptation to changes in environmental conditions and fungal availability [60]. When the carbon invested in the mycorrhizal partner surpasses the host capacity to produce it [58], facultative mycorrhizal plants seem to be capable of “deciding” to cease an alliance that is no longer beneficial [60]. FM species also appear to be favored during the colonization of new habitats such as raw substrates (new volcanic rocks) where nutrients are limited, and in areas outside their native range, where adequate fungi may not be present yet [40, 44, 54].

FM plants are more difficult to identify than OM and NM because the great majority of studies do not include sufficient sampling within a taxon to explore its mycorrhizal status across space and time. At first, this denomination was applied to plant species frequently found with low levels of colonization [8]. However, nowadays it is based on observations of both the presence and absence of mycorrhizal associations in plant roots of individuals of a given species, or even within a given individual at different points in time [12]. The comparison of four databases of mycorrhizal occurrence in Europe and Asia showed discrepancies in the criteria to identify the plants mycorrhizal status [40]. This suggests that many species might be wrongly designated as obligate or non-mycorrhizal depending on the sampling period and the approach followed by the researcher [8]. The combination of field observations and greenhouse experiments on selected taxa is the most promising way to expand our understanding of FM plants [27].

Ferns are among the oldest groups of extant vascular plants, but the diversity, functionality, and dynamics of their alliance with mycorrhizal fungi is a largely ignored aspect of their ecology [30]. Although AMF are observed in fern roots, little is known about the plant dependence on this type of association. Ferns exhibit a lower percentage of mycorrhizal species (66%) than angiosperms (72–80%) and gymnosperms (100%), suggesting that they might rely less on mycorrhizae than other vascular plant groups [9, 26]. The reason behind this discrepancy is still poorly understood, but it could be related to the development of adaptations to survive in new challenging habitats such as the epiphytic and the aquatic [27], and/or the passive incorporation of nutrients during water uptake in ferns [24]. Therefore, we may expect that facultative mycorrhization would be common in this lineage. Nevertheless, previous reports are scarce and they are based on the visual examination of fern roots indicating the presence and absence of AMF in certain species [26, 59]. The study of fern-mycorrhizal associations in a controlled environment is needed to better understand the dynamic of FM partnerships.

*Struthiopteris spicant* (L.) Weiss is a terrestrial fern from the family Blechnaceae, with a disjunct distribution in northwestern North America and across Europe [38]. It occurs in a wide diversity of habitats, ranging from acidic, humus-rich peat soils to well-developed forest soils, and sandy substrates. *Struthiopteris spicant* prefers partial to full shade moist environments, enduring temperatures under − 20 °C [38]. Preliminary field observations suggest a facultative mycorrhizal status in this species because it has been found with and without mycorrhizae (M. Kessler *pers. obs.*).

The current study aimed to assess how nutrient and light limitation influence the association *Struthiopteris spicant* with arbuscular mycorrhizal fungi. We conducted a three-factor (Light, Phosphorus, Nitrogen) greenhouse experiment to test the following hypotheses: (a) *S. spicant* is a facultative mycorrhizal fern species. (b) The presence of arbuscular mycorrhizal fungi in the roots of this species is enhanced by high light and low nutrient availability and restricted under low light and high nutrient conditions.

## Results

Our metabarcoding analysis yielded a total of 1,622,246 reads corresponding to 1798 amplicon sequence variants (ASVs), where 22,831 reads (214 ASVs) belonged to Glomeromycota. In general, we identified 124 fungal species included in 97 genera, 74 families, 39 orders, and eight phyla (see Additional file 1).

**Fig. 1:**
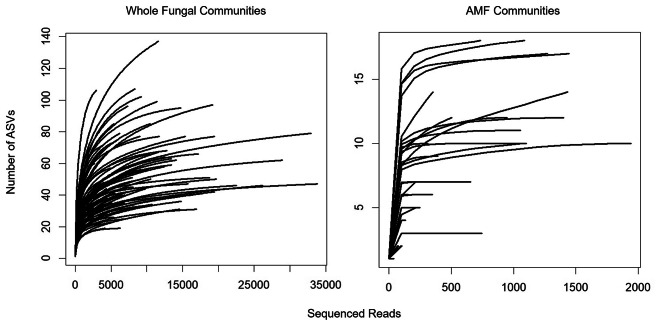
Rarefaction curves representing the expected number of species as a function of the sequenced reads. Legend- Left: whole fungal communities; Right: arbuscular mycorrhizal fungal communities. ASVs- amplicon sequence variants; AMF- arbuscular mycorrhizal fungi. Each individual curve corresponds to a study plant

Rarefaction curves representing ASV number as a function of the sequenced reads per sample did not reach a saturation point in some cases (Fig. 1). However, when considering only the arbuscular mycorrhizal fungi (AMF), most curves showed fast saturation after 200 reads, implying that the sequencing depth was adequate to analyse AMF communities associated with *S. spicant* (Fig. 1).

**Fig. 2:**
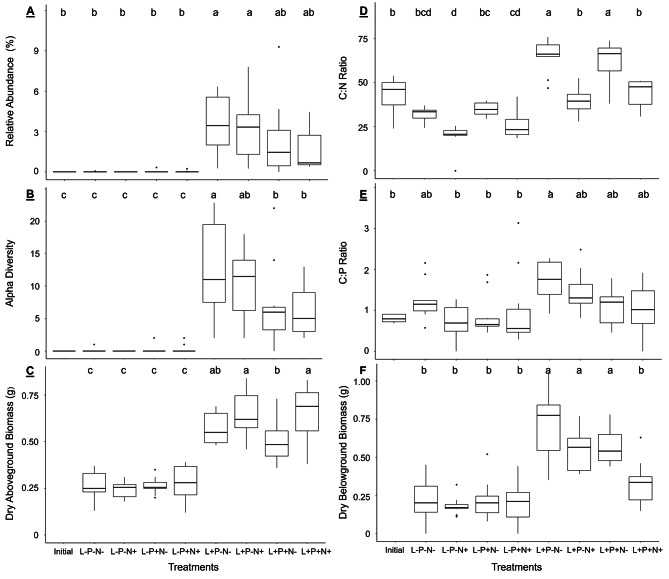
Effect of variations in light, phosphorus, and nitrogen availability on *Strupthiopteris spicant*. Legend- **(A)** Relative abundance (percentage of AMF sequences relative to all fungal sequences of a sample), **(B)** Alfa diversity (number of AMF ASVs per sample), **(C)** Dry aboveground biomass, **(D)** C:N ratio, **(E)** C:P ratio, **(F)** Dry belowground biomass. Different letters indicate significant differences among the treatments calculated using the Games-Howell *post hoc* test (*P* < 0.05). The aboveground and belowground biomasses were not measured during the Initial stage. AMF- arbuscular mycorrhizal fungi, L-light, P-phosphorus, N-nitrogen. Symbols +/- indicate addition/no addition of the correspondent nutrient

The PERMANOVA test showed a significant impact of the factors Light (R² = 0.13289, *p* < 0.01**) and Phosphorus (R² = 0.04713, *p* < 0.01**) on plant growth, nutrient accumulation, and fungal community composition. In general, considering AMF richness (sum of ASVs in a sample) and AMF abundance (abundance of AMF sequences relative to all fungal sequences of a sample), AMF were 10-80% more abundant and diverse in plants receiving high light than in those under low light conditions (Fig. 2A-B).

Although specimens receiving high light and nitrogen (N) produced on average twice as much aboveground biomass than the low light ones, the differences among high light treatments were not significant (Fig. 2C). On the contrary, the lack of fertilization, either P or N, in the presence of high light favored belowground biomass production leading to an increment of more than 20% compared to other treatments (Fig. 2F). Interestingly, plants growing with high light and added nutrients had a similar belowground biomass production to those growing under low light.

Leaf nutrient content also varied between treatments. We observed an increase in the C:N ratio of plants growing with high light and low N (nearly 70 g of carbon (C) per 1 g of nitrogen), whereas the ratio decreased in specimens with low light (approx. 30 g C per 1 g N) despite the availability of nutrients. Finally, we did not observe a clear pattern of differentiation when comparing the C:P ratio among treatments (Fig. 2D-E).

**Fig. 3:**
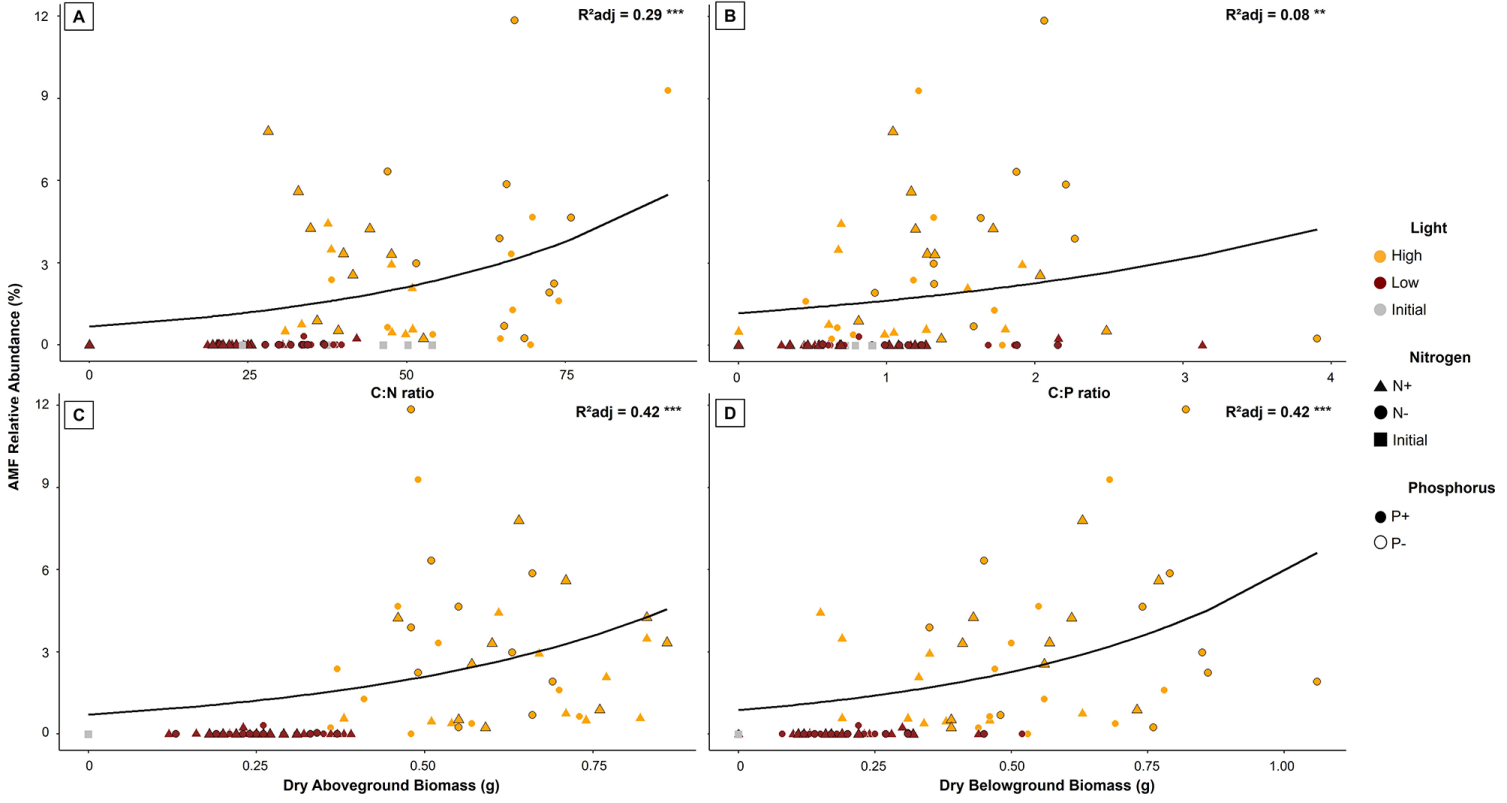
Relationships between the relative abundance of AMF and growth and nutrient use efficiency of *Struthioptheris spicant*. Legend- **(A)** C:N ratio; **(B)** Dry aboveground biomass; **(C)** C:P ratio; **(D)** Dry belowground biomass. Significance levels: ** (*P* < 0.01), *** (*P* < 0.001)

The logarithmic regression analyses revealed positive relationships between AMF abundance and plant growth variables. High light was the main determinant for the presence of Glomeromycota fungi, so that we observed an upward trend of C:N and C:P ratios as well as above- and belowground biomass production when AMF abundance increased under high light (Fig. 3).

**Fig. 4:**
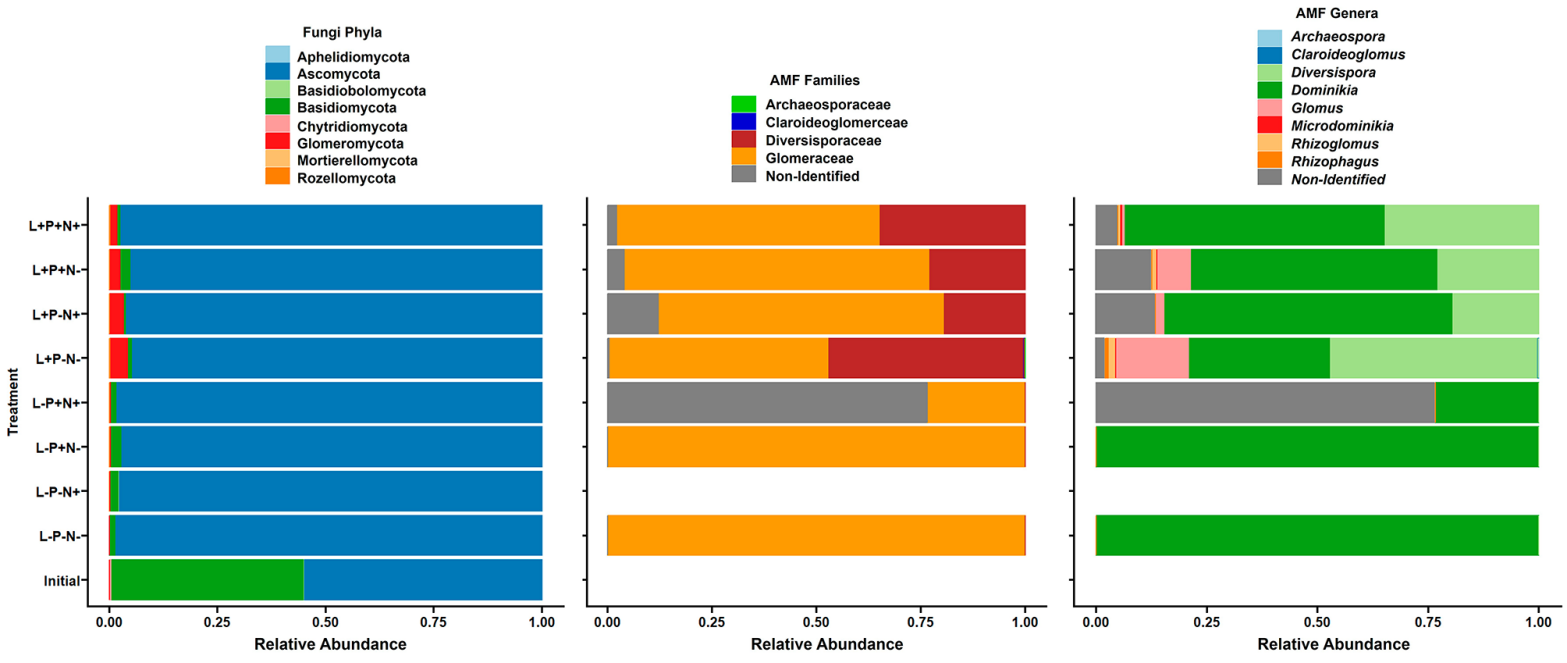
Taxonomic composition of fungal communities associated to *Strupthiopteris spicant* under different light and nutrient conditions. Legend- The initial stage and eight treatments are represented by Initial (Initial stage) and different combinations of L (Light), P (Phosphorus), and N (Nitrogen). Left: Whole fungal communities at phylum level; Centre: AMF communities at family level; Right: AMF communities at genus level

The compositional analysis of the whole fungal communities associated with *S. spicant* roots revealed clear differences among treatments. From an Initial stage (initial conditions) dominated by the phylum Basidiomycota and Ascomycota, we observed that Ascomycota passed to dominate all the communities during the experiment, while Glomeromycota members increased in treatments receiving high light (Fig. 4).

Focusing on the arbuscular mycorrhizal fungi (Glomeromycota), we found an influence of the light quantity and nutrient regimen on the relative abundance of the families and genera. While initial specimens were AMF-free, Glomeraceae was represented in seven of the eight treatments (> 50% on average) and Diversisporaceae showed its highest abundance in plants receiving high light (15-50%) (Fig. 4). Furthermore, the diversity and abundance of AMF genera was greater in high light conditions (*Diversispora*, *Glomus*, *Dominikia* and *Rhizoglomus*) with the genus *Glomus* being more abundant in low nitrogen treatments (Fig. 4).

The Indicator Species Analysis identified the taxa characterizing each of the conditions established during the experiment using the relative abundance and relative frequency of occurrence of species in the treatments. We identified 17 indicator species across treatments. Treatment L2 (P-N+) showed the most distinct community, represented by *Acremonium nepalense* (A), *Dactylonectria amazonica, Ilyonectria macrodidyma, Leucosporidium yakuticum* (B), *Dominikia achra*, and *Dominikia duoreactiva* (Table 1). We found more indicator species for high light availability than for low light, and any of the levels of P and N.

**Table 1:** Indicator taxa associated with *Struthiopteris spicant* under different levels of light, phosphorus and nitrogen addition

Indicator taxa	Light+	Light-	N+	N-	P+	P-
**Ascomycota**						
*Acremonium nepalense* (A)	X		X			X
*Acremonium nepalense* (B)		X				
*Acremonium nepalense* (C)	X			X		
*Cladosporium ramotenellum*	X					
*Dactylonectriaamazonica*						X
*Dactylonectria* sp.	X					
Didymellaceae		X	X			X
*Gliocladium cibotii*	X					
*Ilyonectria macrodidyma*	X		X		X	X
*Penicillium* sp.	X					
*Plectosphaerella niemeijerarum*	X					
*Pseudofabraeacitricarpa*	X					
**Basidiomycota**						
*Curvibasidium* sp.				X	X	
*Leucosporidium yakuticum* (A)					X	
*Leucosporidium yakuticum* (B)	X			X		
**Glomeromycota**						
*Dominikia achra*	X		X			X
*Dominikia duoreactiva*	X		X			X

*Taxa were determined using the Indicator Species Analysis [10]

**Fig. 5:**
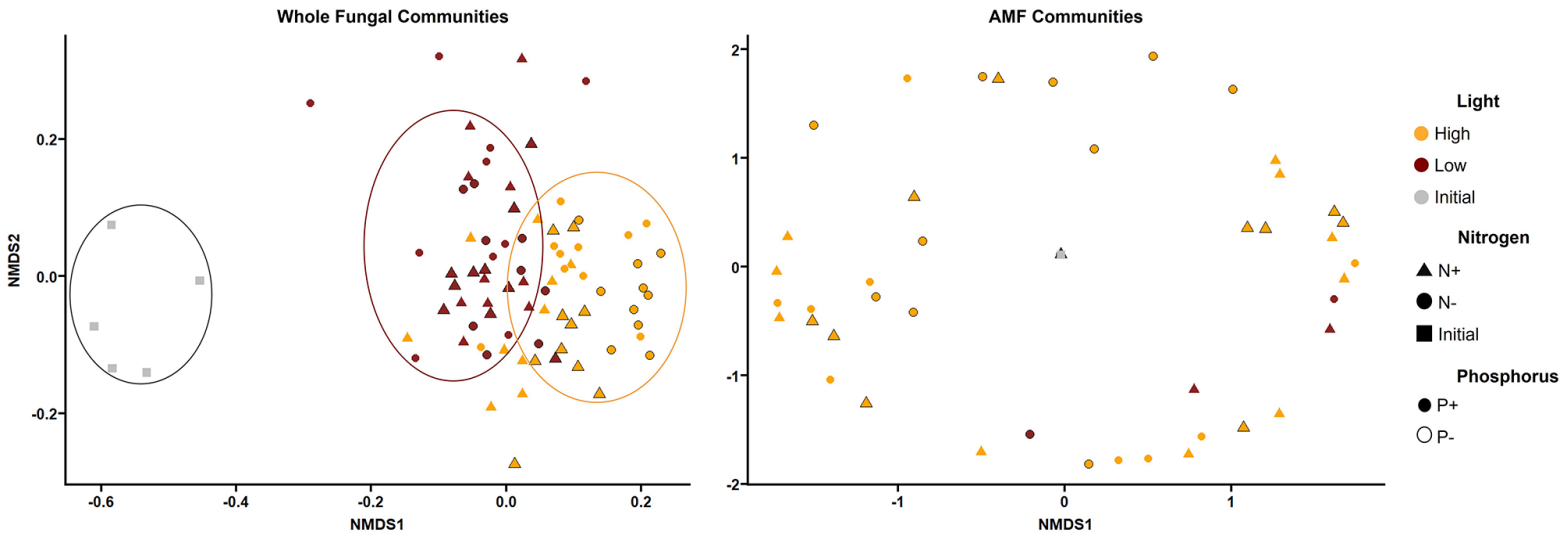
NMDS ordination based on Bray-Curtis distances showing the similarities in fungal community composition among eight treatments. Legend- Left: Whole fungal communities; Right: AMF communities. The initial values correspond to measures taken in five randomly selected plants upon starting the experiment

We used a non-metric multidimensional scaling (NMDS) analysis based on Bray-Curtis distance matrix to visualize differences in fungal community composition among treatments. Our results revealed that fungal communities were differentiated according to the quantity of light received by the host plants (Fig. 5). The cluster formed by the initial samples highlighted the existence of a very distinctive fungal community at the beginning of the experiment. In contrast, there were no evident patterns of similarity among plants when analyzing solely the AMF communities (Fig. 5). The other two factors (N and P) did not explain the fungal community difference in the fern roots.

## Discussion

Mycorrhizal relationships are usually seen as an ecological advantage for plants and a secure carbon source for fungi [8, 54]. However, the mutualism of these associations is dependent on light incidence, nutrient availability, and the genotypes of the plants and fungi [26, 45, 52], and a certain, still unknown proportion of plant species appears to be able to control the occurrence of these associations, thus being facultatively mycorrhizal (FM) [9, 54, 60]. Previous studies analyzing the effects of environmental factors on the arbuscular mycorrhizal fungi (AMF) symbiosis have largely focused on angiosperms, neglecting mycorrhizal relationships in ferns [27, 56]. In the relatively few studies analyzing the presence of fungal symbionts in ferns (e.g., 3,27,29), reports of FM species follow uneven methodological approaches [27]. Our study aimed to explore the nature of FM associations in ferns by evaluating the responses of mycorrhizal fungi associated with *Struthiopteris spicant* to differences in light, phosphorus (P), and nitrogen (N) constraints in a greenhouse experiment.

Facultative mycorrhizal (FM) plants may only associate with AMF when environmental conditions (mainly nutrients and light availability) are adequate [8, 41, 42]. When assessing the results of our experiments, we must consider that the sequencing approach used by us may selectively bias towards certain species and genera [32]. Thus, the differences in relative abundances between species must be viewed with care. Nevertheless, this is the standard method for studying fungal root microbiomes [20, 48, 49], and the differences in relative abundances of each species between treatments are so clear that they are unlikely to be determined by sequencing biases. Furthermore, our study does not provide direct evidence that ferns and the potential AMF partners interact physiologically [27], but it confirms that *S. spicant* can grow with and without arbuscular mycorrhizal fungi. Bearing these caveats in mind, this is the first experimental confirmation of facultative mycorrhization in ferns.

Facultative mycorrhization may be an adaptive strategy to optimize dispersal and growth when ecological conditions and fungal availability fluctuate [60]. To avoid the limitations linked to a dependence on mycorrhizal fungi, ferns might have developed strategies that favor their endurance in new sites where the appropriate fungi are missing. For example, [36] suggested that the evolution of short-lived chlorophyllous spores, which occur in about 15% of all fern species, is an adaptation to avoid dependence on mycorrhizal fungi during spore germination and early development. In the case of *S. spicant*, it is possible that its mycorrhizal status facilitated the colonization of new areas as has been previously reported for FM taxa [37, 40, 43]. However, with the current data, we can only confirm that the FM status in *S. spicant* is driven by the limitation of photosynthesis versus nutrient availability, which is revealed by the observed positive relationship between AMF abundance and plant nutrient use efficiency and growth.

We found light to be the primary factor determining the presence and diversity of the AMF communities in *S. spicant*, coinciding with previous findings in field and greenhouse experiments using angiosperms, where low light conditions restrict plant photosynthesis and therefore carbon production, leading to a decline in AMF richness and abundance [30, 51]. Some authors have argued that this outcome is a consequence of enhanced competition of the fungi for the host carbohydrates, which could cause the exclusion of less adequate taxa [30]. However, our results indicate a striking reduction by more than 90% in AMF presence and diversity in the host roots when it is grown under low light conditions. This drastic change could rather be explained by negative plant-soil feedback.

Plant-soil feedback (PSF) occurs when plants alter the physical, chemical, and/or biotic conditions of the soil, affecting their performance and that of other organisms [4]. Strong negative PSF has been previously reported when light is a limiting element [34, 35] and might be caused by different factors such as soil pathogen proliferation [47], parasitic mycorrhizal fungus associations [22, 52], and the influence of soil nutrient availability [2]. The latter is one of the most studied factors in relation to plant-AMF relationships (e.g., [13, 16, 41, 42]) [5]. studied the effects of P and N limitation on the arbuscular mycorrhizal symbiosis in *Medicago truncatula* and found that an increase in mycorrhizae formation was linked to systemic signaling by the plant nutrient status. Low P availability induces a physiological state in plants that favors root colonization [53], whereas high P can inhibit AMF vesicles and entry point formation, reducing also the length of external hyphae [21].

Concordantly, we observed an influence of phosphorus availability on the relative abundance and alpha diversity of AMF, with both parameters increasing when phosphorus was limited. The crucial role of mycorrhizal fungi in soil phosphorus mobilization and uptake has been largely studied in angiosperms and it is considered one of the main positive effects of AMF symbiosis [8, 22]. Finding a rise in AMF presence within *S. spicant* roots when specimens grow in low soil phosphorus concentrations, confirms the same role of AMF in ferns. On the other hand, we did not encounter any clear effect of nitrogen fertilization on the fungal communities, although there was a marked impact on above-ground biomass.

During our experiment, the remarkable influence of light on plant development surpassed the nutrient effect. Specimens receiving low light produced 50% less biomass than those receiving high light, regardless of the quantity of phosphorus and nitrogen available. Growing in low light conditions can limit plant growth by weakening their photosynthetic mechanism and even altering the chloroplast ultrastructure. If the light supply is insufficient, the production of photosynthetically fixed carbon is affected [57, 58]. Consequently, biomass production and allocation change, and both plant growth and carbon storage might be reduced. Under these circumstances, there is also a reduction in mycorrhizal colonization. When light is limited, the plant reduces its investment in the association [2, 24] because the C drain to the fungus exceeds the benefits obtained in terms of phosphorus and nitrogen acquisition [6, 42, 59]. This resource drop might reduce AMF diversity due to the preferential allocation of carbon to the most beneficial fungal partners [42] or even, in the case of FM plants, terminate the symbiosis. In ecosystems where light is not a limiting factor, plant growth is restricted by AMF [8] and nutrient availability. Plants are capable of optimizing their resource acquisition, keeping an equilibrium between above- and belowground production [57, 58]. Our results corroborated previous data: specimens growing in high light showed the maximum root development when they were limited by phosphorus and nitrogen [17].

Moreover, AMF relative abundance exhibited a direct positive influence on the growth and nutrient use efficiency of *S. spicant*, indicated by a boost in biomass production and a rise of C:N and C:P ratios. This shows that despite the additional N and P likely provided by the fungi, plants grown under high light made more optimal use of nitrogen and phosphorus than plants under low light conditions [64]. In the first case, growth may have been limited more by nutrient availability, whereas in the second carbon was probably the restrictive factor.

Focusing on the taxonomic composition of the fungal communities linked to *S. spicant* roots, we found a dominance of Ascomycota, Basidiomycota, and Glomeromycota, which coincides with previous reports in ferns [3, 18, 49, 60]. The structure and functionality of root-associated fungal communities are shaped by the host characteristics and abiotic factors such as the pH, soil enzyme activity, light incidence, and nutrients availability [29] The establishment of Ascomycota as the dominant group might be related to the change of soil at the onset of the experiment, or its higher adaptive capacity to benefit from environmental changes, as indicated by [31].

Glomeraceae has been recognized as the most common AMF family at a global level [43], as is also the case in our study. Additionally, we found a significant contribution of Diversisporaceae in plants subjected to the high light treatments. Whether this outcome reflects a positive effect of high light for this family or a shift in competitive interactions with other fungi under low light is still unclear. However, the differences in the relative abundance of this family among high light treatments might be connected to nutrient availability. Diversisporaceae was particularly abundant when neither P nor N was added to the soil, contrasting with previous findings for angiosperms at phosphorous-rich sites [15]. These conflicting results may reflect the influence of numerous factors such as soil conditions, natural versus experimental conditions, and the plant taxa involved.

The difference in AMF community composition between the high and low light treatments is even more striking at the genus level, with the appearance of *Glomus, Dominikia* and *Rhizoglomus*, and the explosion of *Diversispora* abundance in specimens receiving high light. The predominance of the genus *Dominikia* in AMF communities associated with ferns was previously reported by [20] in a metagenomic study including 12 fern and lycophyte species.

Finally, we identified shared and specific species characterizing all the abiotic conditions included in our experiment and found that most fungi thrive in well-illuminated and low-phosphorus environments (Table 1). Finding unique fungal communities across treatments emphasizes the sensitivity of plant-fungus associations to changes in light and nutrient availability [6].

The results of this study should be carefully interpreted since they respond to the intensity levels established for each factor (light, nitrogen, phosphorous), and different outcomes might be obtained with different parameter settings. Still, this combination of variables to evaluate the response of a single fern species to mycorrhization provides a better understanding of fern-AMF interactions than field studies, revealing responses that are independent of environmental variations or plant community shifts [52]. This study is the first to investigate the importance of light and nutrient availability in determining fern-AMF relationships.

We confirmed that *Struthiopteris spicant* is a facultative mycorrhizal species that responds very sensitively to changes in light and nutrient conditions, leading to shifts in the composition and diversity of the associated mycorrhizal communities. However, whether all ferns respond equally to environmental variations, or if they are less nutrient-dependent than angiosperms, as hypothesized by [27], can only be verified by direct comparative studies of their ecophysiology.

## Conclusions

In this study we assessed the influence of nutrient and light availability on the association of *Struthiopteris spicant* with arbuscular mycorrhizal fungi, revealing the major importance of light for fungal community differentiation and plant productivity. High light conditions positively influenced fungal community composition, plant biomass, and nutrient accumulation accounting for an increment of 10–80% in each variable. Moreover, the effect of phosphorus and nitrogen content seemed to be determined also by the light incidence and probably the fungal genotype. The genera *Diversispora*, *Glomus*, *Dominikia* and *Rhizoglomus* were mainly present under high light conditions whereas the species *Dominikia achra* and *Dominikia duoreactiva* associated to the studied fern roots in an environment with high light, low P, and high N availability. We conclude that *S. spicant* is a facultative mycorrhizal fern and future efforts should be directed to investigate its natural populations under diverse environmental conditions to better understand the dynamics of facultatively mycorrhizal ferns and their arbuscular mycorrhizal fungi associations.

## Methods

### Experimental design

We intended to evaluate the influence of light and nutrient availability on the interaction of *Struthiopteris spicant* with arbuscular mycorrhizal fungi (AMF). The high tolerance of this species to changes in pH, soil, and light conditions made it the perfect candidate for our experiment. To accomplish test our hypotheses we measured three parameters: plant development, AMF identity and relative abundance, and leaf nutrient content.

We started conducting a four months pre-experiment (November 2020 to March 2021) to define the proportions of light, phosphorus (P), and nitrogen (N) that were adequate for our study (see Additional file 2)’. Keeping a low soil pH level and a particular watering frequency resulted to be critical for plant survival.

**Fig. 6:**
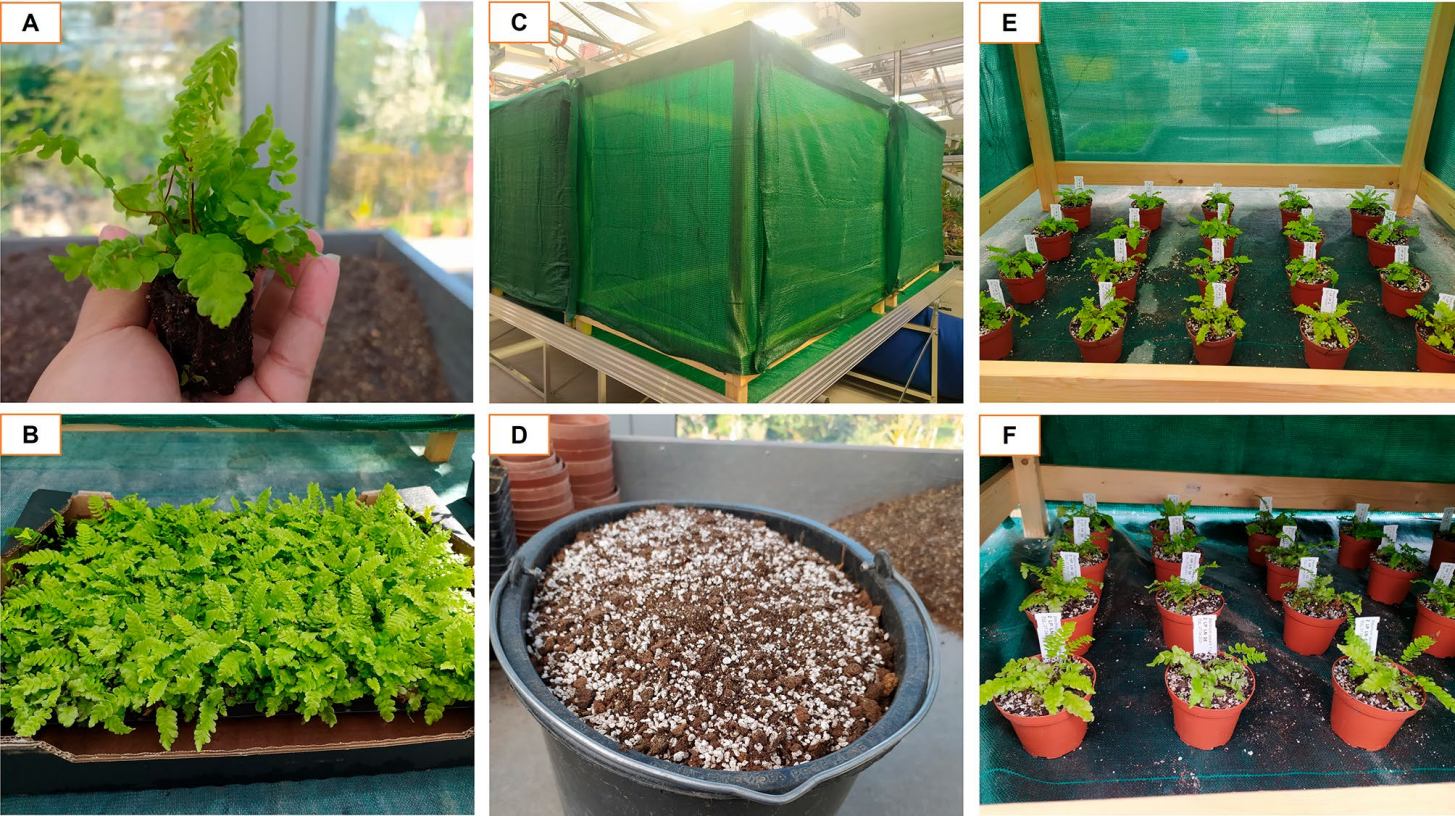
Greenhouse experiment setup. Legend- **(A)** Initial size of the sporophytes; **(B)** Original plant container; **(C)** Experimental containers; **(D)** Substrate utilized; **(E)** Treatments L1-4 (1 shade cloth layer); **(F)** Treatments Sh-4 (3 shade cloth layers)

The final greenhouse experiment was carried out from May to August 2021 at the research greenhouses of Zurich Botanical Garden. We selected 80 young fully developed sporophytes grown under identical conditions at a commercial nursery (Farnwerk, Härkingen, Switzerland) (Fig. 6A-B). Before transplanting them into 300 ml individual pots filled with low-nutrient substrate, we carefully washed their roots with tap water to eliminate the soil grains.

To gather the initial data per specimen we collected one leaf for the nutrient analysis and 10 root fragments for fungal DNA sequencing and placed them in labeled paper bags with silica gel. We also assessed the initial number of leaves and measured the length of the three longest ones. Plants were kept under controlled conditions of temperature (12.8°-18.8 °C) and relative humidity (50–60%) in 1 × 1 m² wooden structures covered by shade cloth (Fig. 6C).

The substrate utilized during this experiment was a 9:1 blend of a low-nutrient soil mixture (2 parts peat: 1 part perlite: 1 part quartz sand) and soil collected at three localities where *S. spicant* grows in the wild (Fig. 6D). Given the high pH of this substrate (pH = 7.58) and the preferential pH range of 5.0-5.8 for *S. spicant* soils and AMF colonization [49], we prepared a solution dissolving 25 milligrams of tannin soluble powder in 10 L of rainwater to lower the soil pH. Plants were watered twice each week with this acidic solution and common rainwater, respectively. We monitored the soil pH using a Growline pH Meter (Hanna Instruments Deutschland GmbH, Germany), following the manufacturer’s instructions. To provide the necessary micro- and macronutrients, all the specimens received 75 ml of a general nutrient solution every two weeks (see Additional file 3).

We exposed the plants to 8 different treatments featuring two levels of phosphorus, nitrogen, and light availability, for a fully factorial three-level design (Table 2). Each treatment included 10 replicates for a total of 80 individuals. Every four weeks, we randomly rearranged the plant positions, and recorded the number of leaves and the length of the three longest leaves per specimen.

#### Light availability

We first measured the light received by individuals of *S. spicant* at a natural population on a sunny day from 11:00 am to 12:30 pm, to assess natural light conditions. Values ranged from 395 µmol m^-2^ s^-1^ in open areas of the forest to 11 µmol m.^2^ s.^1^ in deep shade. During the experiment, we controlled light availability using artificial illumination in a 12:12 light-dark cycle and a permeable green shade cloth (shading 63%, 150 cm wide) as cover. The High light category included one layer of shade cloth that allowed the incidence of 58 µmol m.^2^ s.^1^ (Fig. 6E), whereas the category Low consisted of three layers of the same cover with an incidence of 12 µmol m.^2^ s.^1^ (Fig. 6F). Light measurements were taken using a Light Meter (Li – 250 A, LI-COR Biosciences GmbH, Germany).

#### Nutrient availability

The High categories of nutrient availability were defined by the addition of 5 ml of phosphate-based (10 mM KH_2_PO_4_) and nitrate-based (50 mM Ca(NO_3_)_2_) solutions to the corresponding plants every two weeks (see Additional file 3). The Low category did not receive additional nutrients.

### Plant harvest

After 12 weeks of treatment, each plant was separated from its substrate and rinsed with rainwater to eliminate the soil. Approximately 50 mg of roots were collected for fungal DNA extraction and a leaf sample for nutrient analysis. Samples were placed in labeled paper bags with silica gel for their rapid desiccation. The remaining leaves and roots were separated in paper bags, air dried for several weeks, then dried at 70 °C for 40 min, and weighted to measure the above- and belowground biomass (see Additional file 4).

### Leaf nutrient analysis

Leaf samples and five randomly selected initial samples were analyzed at the Albrecht von Haller Institute for Plant Sciences, University of Göttingen, Germany. The total organic carbon (C) and nitrogen (N) concentrations of the samples were determined by a C/N elemental analyser (Vario EL III, Hanau, Germany). The concentration of phosphorus (P) was analysed by the ICP-OES technique (inductively coupled plasma optical emission spectrometry, iCAP 7000, Thermo Fisher Scientific, Germany) after digestion of the material with 65% HNO3 at 195 °C for 8 h. Sample 2LPHN3 was damaged and excluded.

### Molecular analysis

To characterize the fungal communities associated with *S. spicant*, we targeted their ITS rRNA region with universal fungal primers. DNA extraction and purification were performed following the protocol detailed by [20] for ferns and lycophytes. Briefly, approximately 50 mg of roots from each plant were used for DNA extraction with the DNeasy Plant Mini Kit, following the manufacturer’s Quick-Start Protocol (QIAGEN, Hilden, Germany), adapted by [20]. We purified the resulting samples with Monarch Genomic DNA Purification Kit (New England Biolabs, Frankfurt am Main, Germany). The purified genetic material was amplified using KAPA HiFi HotStart ReadyMix (Kapa Biosystems, Wilmington, MA) and primers containing Illumina adapter overhang nucleotide sequences: ITS1F (5’ TCGTCGGCAGC GTCAGATGTGTATAAGAGACAG-CTTGGTCATTTAGAGGAAGTAA) [19]/ ITS4 (5’ GTCTCGTGGGCTCGGAGATGTGTATAAGAGA-CAGTC CTCCGCTTATGATATGC) [61]. The resulting fragments were 450–580 bp length.

**Table 2:** Treatments implemented in the greenhouse experiment using *Struthiopteris spicant*

Treatment	Light Availability	Phosphorus Availability	Nitrogen Availability
L + P-N-	High	Low	Low
L + P-N+	High	Low	High
L + P + N-	High	High	Low
L + P + N+	High	High	High
L-P-N-	Low	Low	Low
L-P-N+	Low	Low	High
L-P + N-	Low	High	Low
L-P + N+	Low	High	High

The sequencing was carried out by EzBiome (Gaithersburg, MD, USA). Libraries were normalized with the Mag-Bind® EquiPure Library Normalization Kit (OmegaBio-tek, Norcross, GA). The pooled libraries were examined utilizing an Agilent 2200 TapeStation and sequenced (2 × 300 bp paired-end read setting) on the MiSeq (Illumina, San Diego, CA). Sample 2LPLN6 failed the Quality Control analysis and therefore was excluded.

Demultiplexed paired-end reads were processed using the package dada2 [11]; v1.22.0) in *R* (v4.1.3). Briefly, we checked for the presence of primers and adaptors and removed them from the sequencing reads using Cutadapt [33]; v4.3). We filtered them by sequence quality discarding those with expected errors greater than 2 and a length under 450 bases. The paired reads were dereplicated and merged with a minimum overlap of 4 bases and a maximum mismatch of 2 bases to build an amplicon sequence variant (ASV) table. We utilized the Naïve bayesian classifier (RDP classifier) with the curated fungal reference sequences from UNITE version 8.3 [1] to identify the obtained ASVs and filter out the sequences that remained unidentified at the phylum level. For taxonomic nomenclature, we followed the classification of the kingdom Fungi proposed by [63].

### Statistical analyses

All the statistical analyses were carried out in *R* (v4.1.3). To assess how the diversity of fungi increased as a function of our sampling effort, we plotted rarefaction curves for the whole fungal communities and the Glomeromycota communities (AMF) by sample. The relative composition of these communities at phylum, family, and genus levels was summarized using the package ggplot [62].

To detect statistical differences in AMF richness (sum of ASVs in a sample) and relative abundance (AMF sequences relative to all fungal sequences of a sample), above- and belowground plant biomass, and leaf nutrient content among treatments, we performed Kruskal-Wallis analyses. The data were previously tested for normality using the Shapiro–Wilk test and for homogeneity of variances using Bartlett’s test. The results indicated a violation of the assumptions of conventional ANOVA, so that we carried out a Kruskal-Wallis analysis complemented by a Games-Howell *post hoc* test (rstatix package).

A PERMANOVA (Permutation Multivariate Analysis of Variance) was performed to assess the role of light quantity, and P and N addition on the fungal community composition, plant growth, and nutrient accumulation, applying the adonis function in the vegan package (v.2.6.4, [44]) with Bray–Curtis dissimilarity. Next, an Indicator Species Analysis allowed us to identify the taxa associated with each of the conditions established during the experiment. The statistical test carried out 9999 permutations using the r.g function in the indicspecies package (v.1.7.12, [14]).

We used non-metric multidimensional scaling (NMDS) to visualize fungal community similarities among plant specimens, considering the influence of light, P, and N levels (Bray–Curtis as distance measure). Finally, we performed a logarithmic regression analysis to assess the relationship between the relative abundance of AMF obtained, and the plant growth and nutrient content variables.

### Electronic supplementary material

Below is the link to the electronic supplementary material.

Supplementary Material 1

Supplementary Material 2

Supplementary Material 3

Supplementary Material 4

## Data Availability

All the data obtained during this study have been deposited in the National Center for Biotechnology Information (NCBI) Sequence Read Archive (SRA) database with BioProject Accession number PRJNA943118. All dataset supporting the conclusions of this article are included within the article and its additional files.
