# Supplementary material for: Facultative mycorrhization in a fern (Struthiopteris spicant L. Weiss) is bound to light intensity
Source: BMC Plant Biol. 2024 Feb 9;24:103. doi: 10.1186/s12870-024-04782-6 (PMC10854079; doi:10.1186/s12870-024-04782-6)
Supplement: Supplementary file 2 — Supplementary Material 2 [file 12870_2024_4782_MOESM2_ESM.docx]

Additional file 2. Details of the pre-experiment design using *Struthiopteris spicant* L. Weiss.

| **Aspects** | **Data** |
| --- | --- |
| Plants | 36 |
| Days | 83 |
| Light level | Without cover  One layer cover  Two layers cover  3 layers cover |
| KH2PO4  solution  concentration | 0.01Mm  0.1Mm  1Mm |
| Ca(NO3)2 solution  concentration | 1Mm  3Mm  5Mm |
