# Supplementary material for: Facultative mycorrhization in a fern (Struthiopteris spicant L. Weiss) is bound to light intensity
Source: BMC Plant Biol. 2024 Feb 9;24:103. doi: 10.1186/s12870-024-04782-6 (PMC10854079; doi:10.1186/s12870-024-04782-6)
Supplement: Supplementary file 3 — Supplementary Material 3 [file 12870_2024_4782_MOESM3_ESM.docx]

Additional file 3. Detailed quantities of salts used to prepare the nutrient solutions used during this study.

| **Solutions** | **Salts** | **Amount to dissolve in 500ml dH2O** | **Volume for 20L solution** |
| --- | --- | --- | --- |
| General nutrient solution | H3BO4 | 4.34g | 20ml |
|  | MnCl2 4H2O | 2.77g |  |
|  | CuSO4 | 0.125g |  |
|  | ZnSO4 7H2O | 0.288g |  |
|  | Na2Mo4 2H2O | 0.048g |  |
|  | NaCl | 0.58g |  |
|  | CoCl2 6H2O | 0.0024g |  |
|  | FeEDTA  -FeSO4 7H2O  -EDTA | 2.78g  3.72g | 50ml |
|  | MgSO4 7H2O | 123.24g | 40ml |
|  | K2SO4 | 87.13g | 150ml |
|  | CaCl2 | 73.51g | 40 |
| Phosphorus (10Mm) | KH2PO4 | 680.4mg | ----------------- |
| Nitrogen (50Mm) | Ca(NO3)2 | 820.45mg | ------------------ |
